# Supplementary material for: Electromechanical oscillations in bilayer graphene
Source: Nat Commun. 2015 Oct 20;6:8582. doi: 10.1038/ncomms9582 (PMC4634209; doi:10.1038/ncomms9582)
Supplement: Supplementary Information — Supplementary Figures 1-11, Supplementary Table 1, Supplementary Notes 1-8 and Supplementary References [file ncomms9582-s1.pdf]

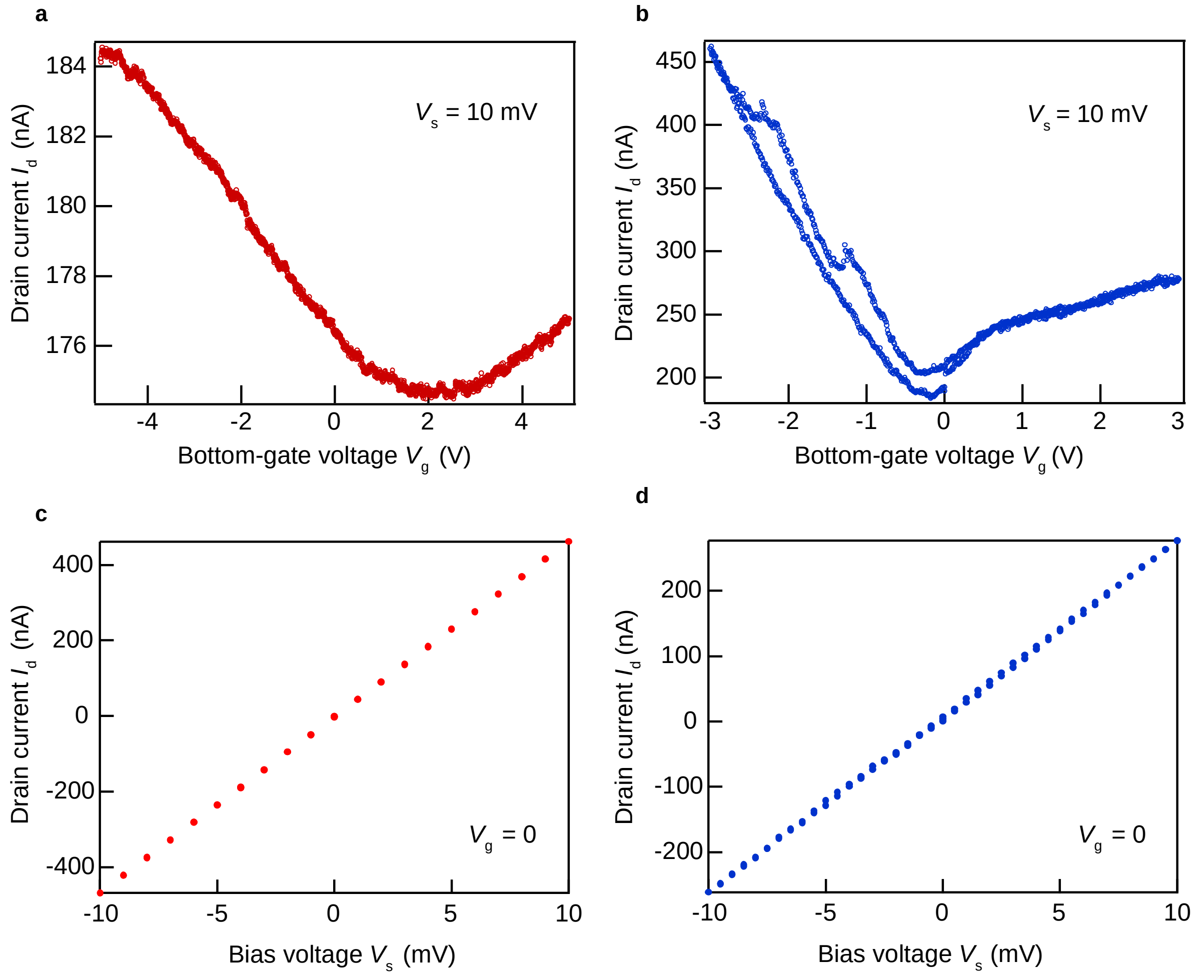

Supplementary Figure 1. Representative transport characteristics of suspended graphene nanoribbons (GNR). **(a-b)** Transfer characteristics of **a,** a single layer GNR (device #1) and **b,** a bilayer GNR (device #6). $I_{ds} - V_{bg}$ curves demonstrate ambipolar behavior with the charge neutrality point V_{CN} at 2V and at 0 respectively. **(c-d)** Output characteristics of the same **c,** single layer device and **d,** bilayer device. The linear $I_{ds} - V_{ds}$ characteristics are indication of ohmic contact.

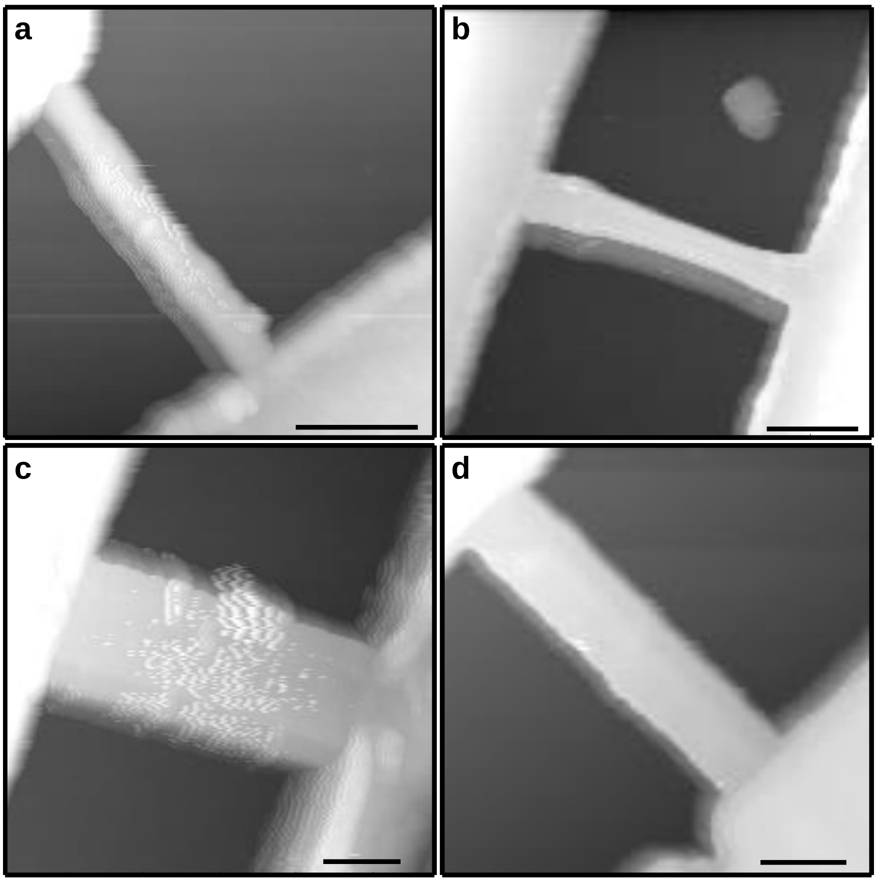

Supplementary Figure 2. Representative AFM images of suspended **a** and **b**, single layer. **c** and **d**, bilayer graphene devices. Scale bar is 200 nm.

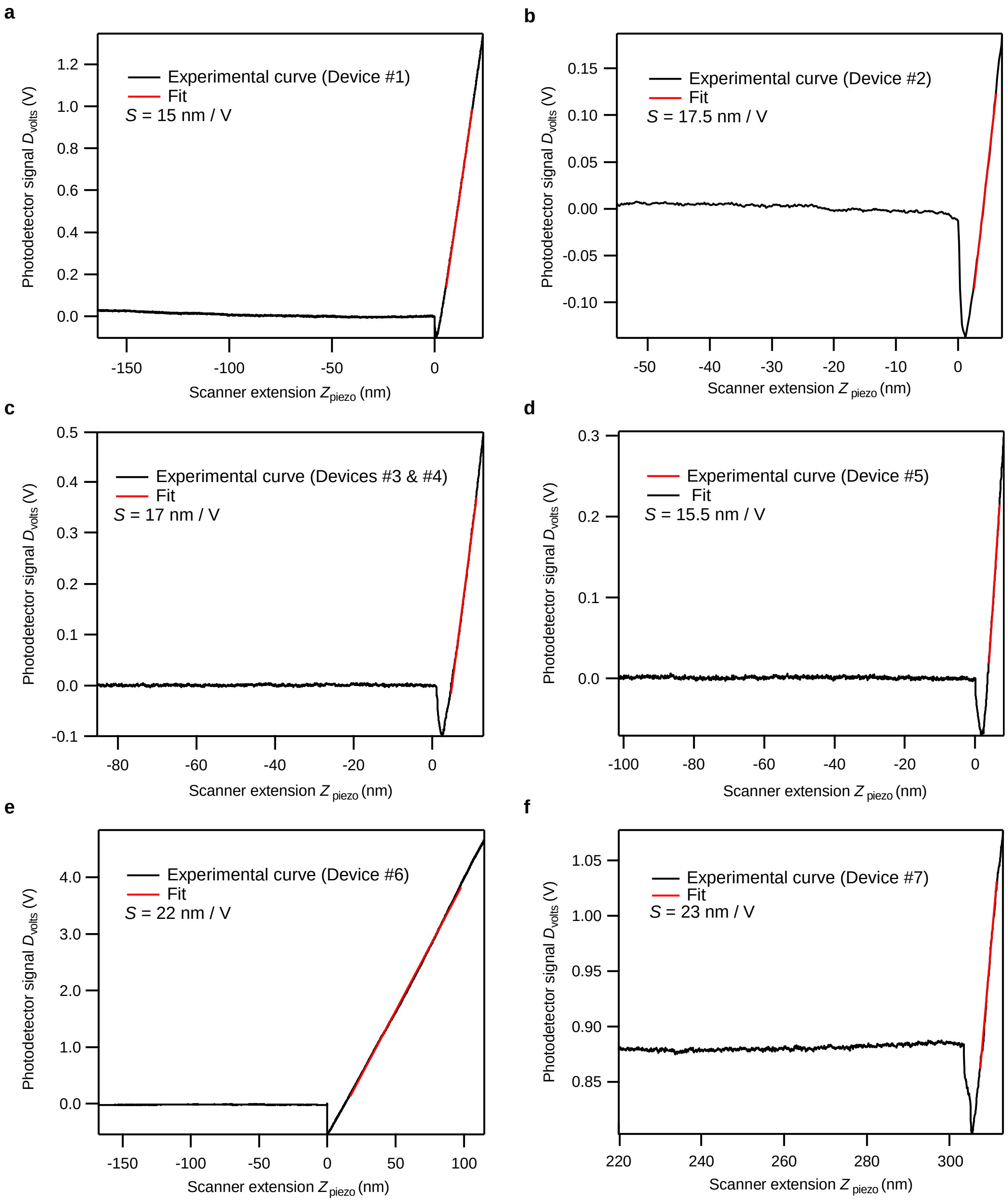

Supplementary Figure 3. Photodetector calibration curves and the calculated photodetector sensitivity for **a,** Device#1. **b,** Device#2. **c,** Device#3 and Device#4 (both measured under the same calibration). **d,** Device#5. **e,** Device#6. **f,** Device#7. Using the calculated sensitivity values the cantilever deflection is determined in nanometers.

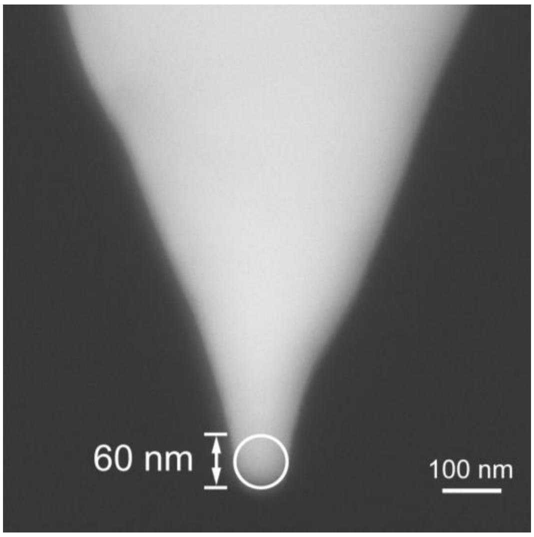

Supplementary Figure 4. Measurement of AFM tip radius using scanning electron microscopy. Magnified SEM image of the AFM probe. The diameter of the tip is approximately 60 nm.

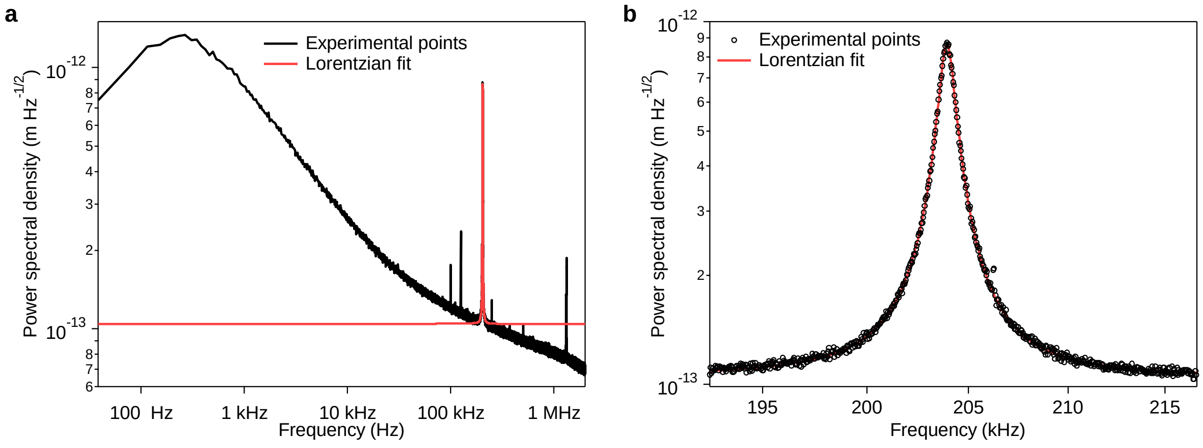

Supplementary Figure 5. **a,** Power spectrum of thermally induced cantilever motion. **b,** The resonance peak fit by a Lorentzian function allowing extraction of the cantilever spring constant

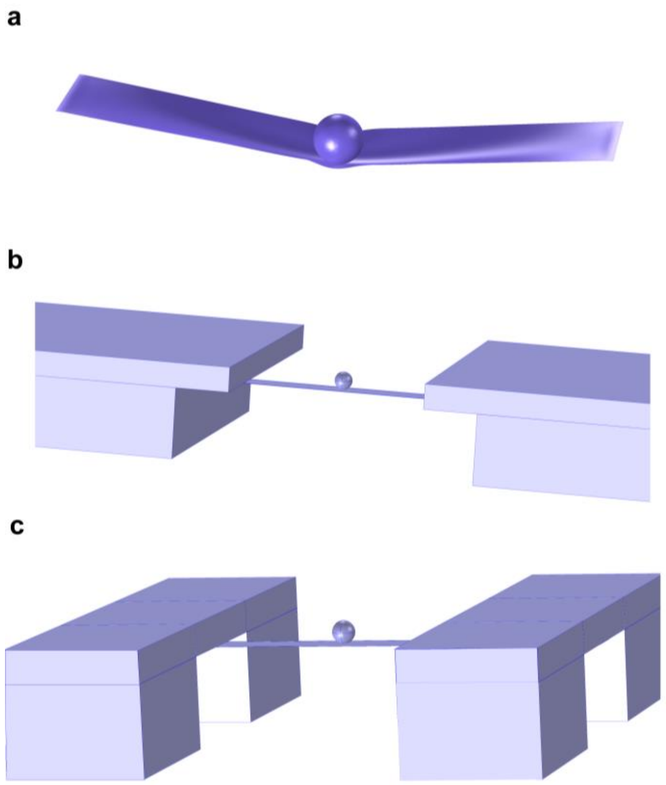

Supplementary Figure 6. FEM simulation of the nanoindentation experiment using various geometries. **a,** Geometry I: the GNR is clamped at the two ends by imposing zero displacement boundary conditions. **b,** Geometry II**:** the GNR is clamped at the two ends by means of two metallic clamps which are partially suspended due the isotropic nature of etching. **c,** Geometry III: the GNR and the clamping region are all suspended. This geometry considers that the interface between graphene and SiO_2 absorbs the HF very fast which leads to the undercut also in the clamped part of the GNR.

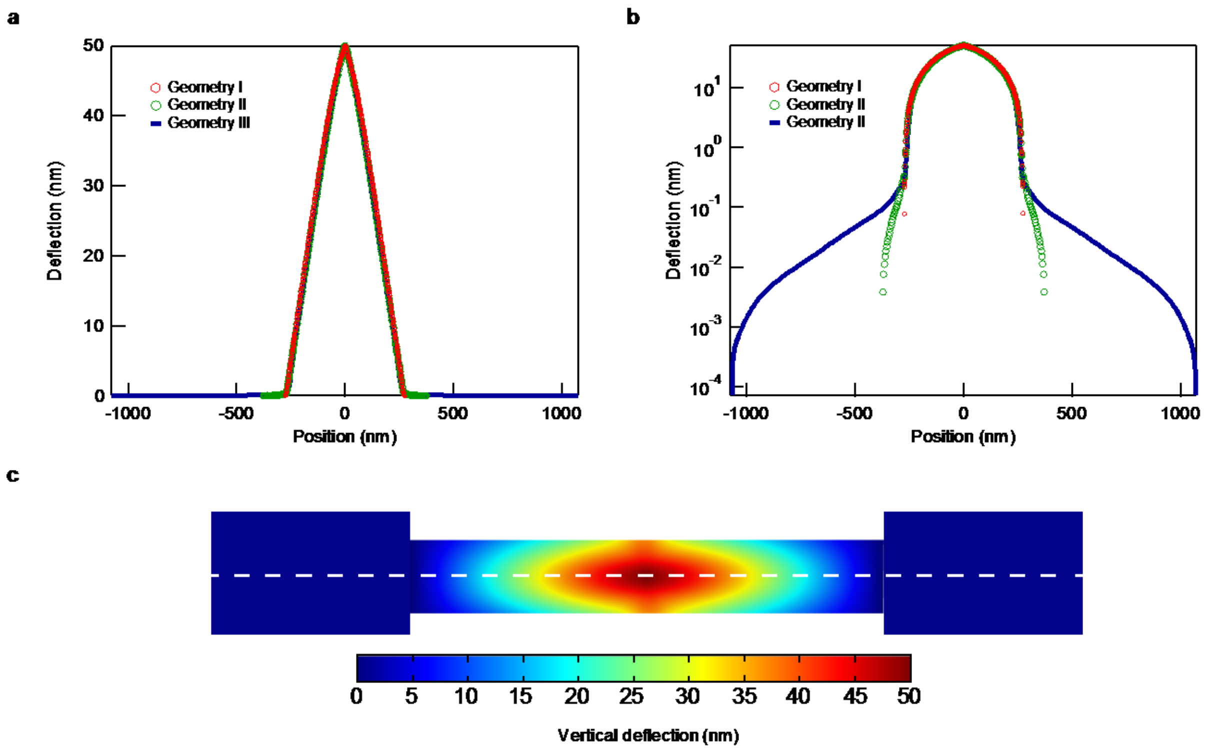

Supplementary Figure 7. Effect of various clamping geometries on the deflection profile of the GNR and the contacts. **a,** linear **b,** logarithmic scale deflection profile along the dashed line shown on **c**.

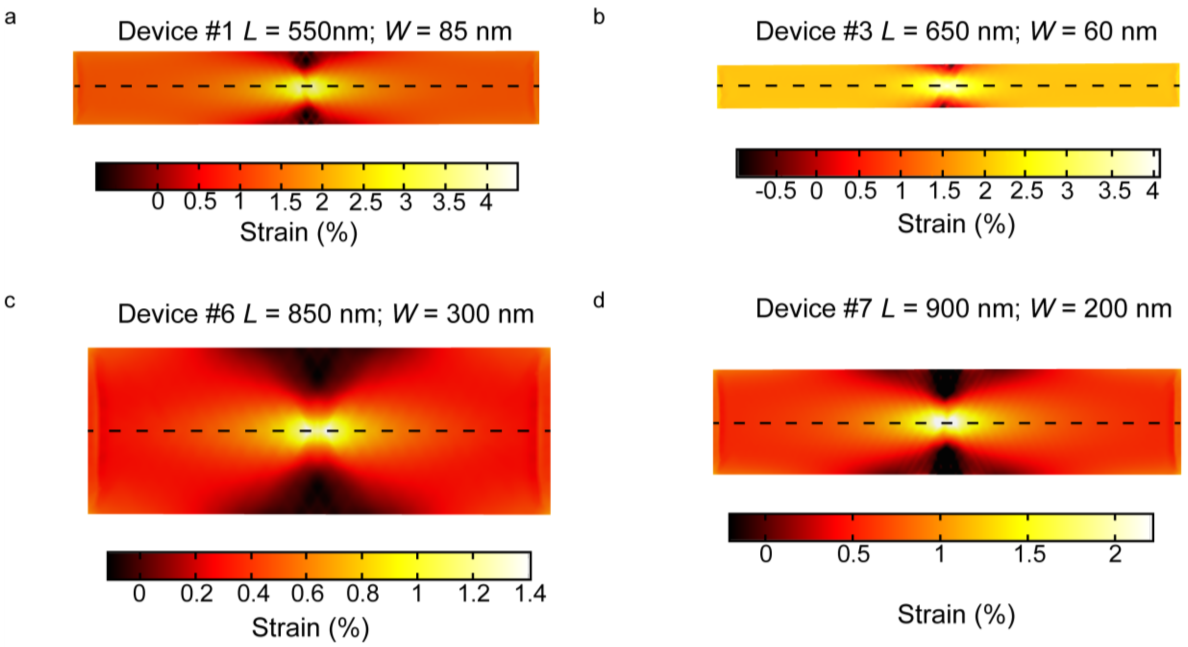

Supplementary Figure 8. 2D map of strain distribution in suspended GNRs in **a,** Device#1. **b,** Device#3. **c,** Device#6 **d,** Device#7. The areas near the AFM tip experience the highest levels of strain due to the local deformation under the AFM tip. Moving away from the center of GNR towards the edges, the effect of local strain is replaced with the contribution from the global deformation of the GNR. The global deformation of GNR leads to the uniform distribution of strain due to the elongation of the GNR.

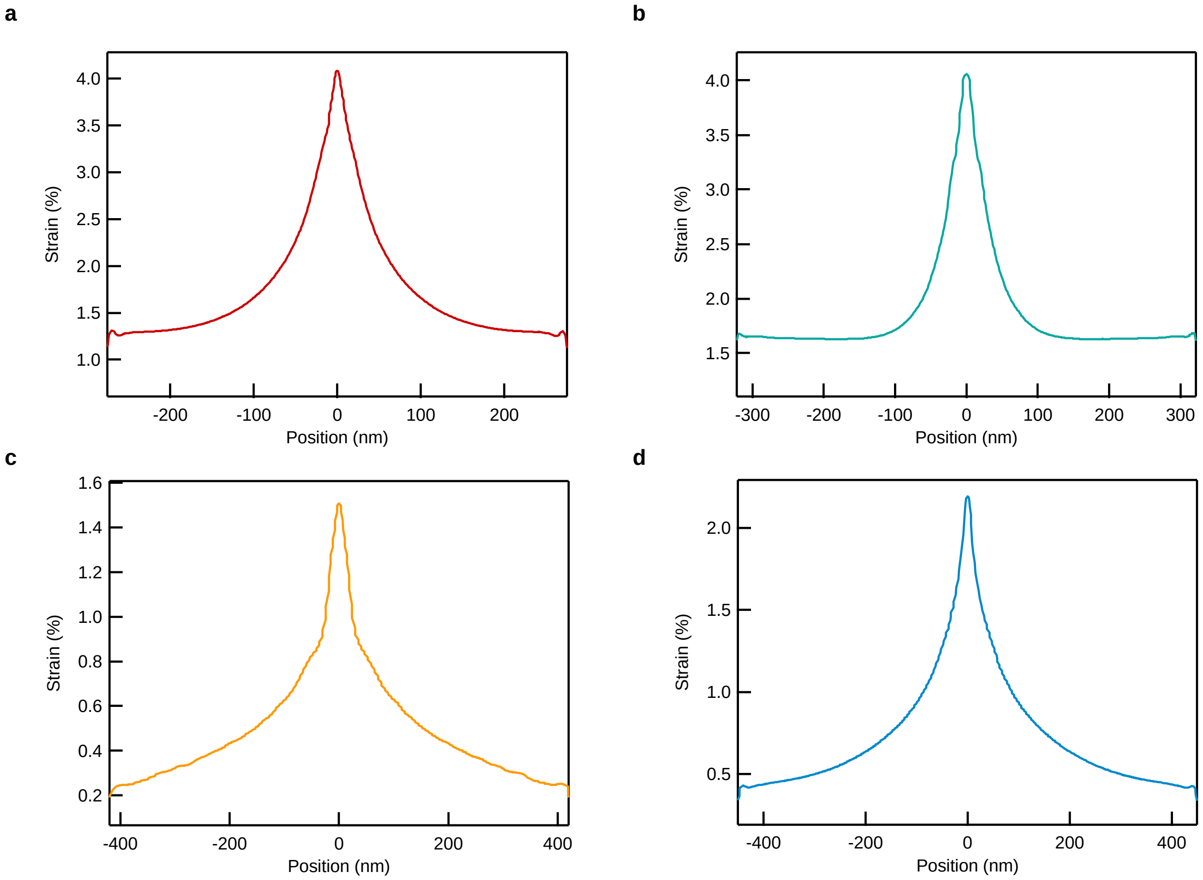

Supplementary Figure 9. Strain profile along the dashed lines in Supplementary Figure 8. **a,** Device #1. **b,** Device #3. **c,** Device #6 **d,** Device #7.

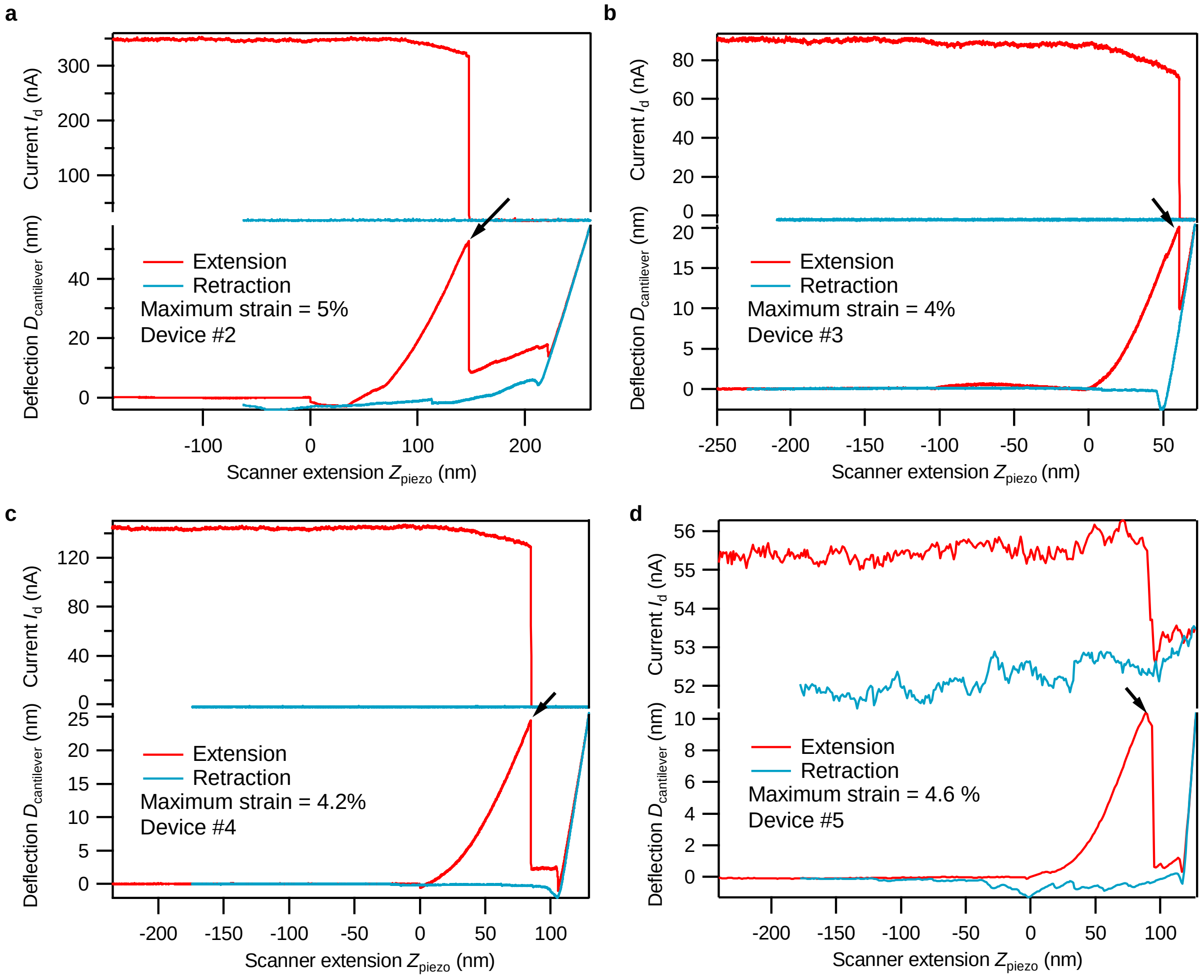

Supplementary Figure 10. Mechanical failure and highest achieved strain for **a,** Device #2. **b,** Device #3. **c,** Device #4. **d,** Device #5. The black arrow shows the point at which mechanical failure occurs.

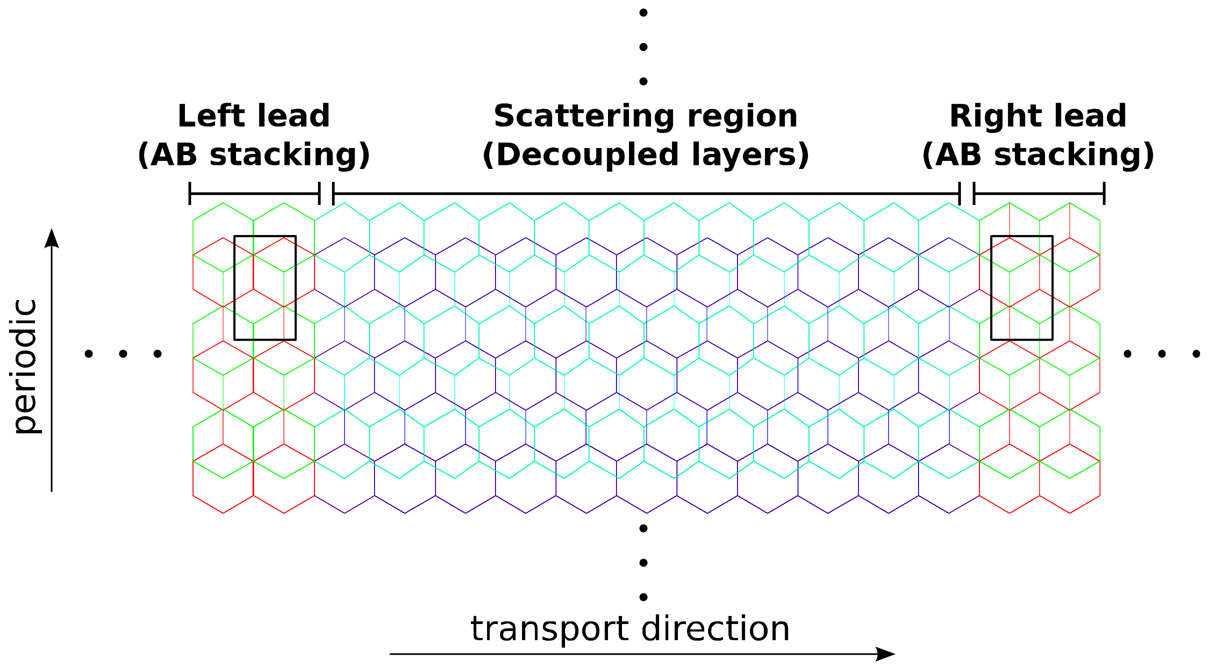

Supplementary Figure 11. Schematic drawing of one of the investigated models ($\Delta W = a$). The two graphene layers are distinguished by color. Black rectangles show the rectangular repeat cell of AB-stacking bilayer graphene leads.

Supplementary Table 1

Device geometry and preparation method

Device number	Width [nm]	Length [nm]	Channel thickness	Preparation method
1	85	550	1L	Exfoliation
2	270	800	1L	CVD
3	60	650	1L	CVD
4	100	700	1L	CVD
5	80	850	1L	CVD
6	300	850	2L	Exfoliation
7	200	900	2L	Exfoliation

Supplementary Note 1

Imaging of suspended GNRs. Special care needs to be taken when imaging suspended graphene devices. Tapping mode operated AFM at low scanning speed (~ 6 μm/s) and scanning direction parallel to the ribbon, allows precise localization of the suspended GNR devices and reduces the risk of mechanical damage. AFM chamber is kept at a constant temperature of ~32°C to minimize thermal drift. Once the suspended GNR is localized, several imaging scans are performed to monitor sample drift. The nanoindentation of the suspended GNR is performed for vanishing drifts, ensuring a strict vertical and minimum lateral deformation. During the experiment, the GNR is biased with an AC signal with an RMS amplitude of 4 mV, a frequency of 8 kHz and the current flowing through it is monitored using a lock-in amplifier. We maintain the back-gate voltage at 0, $V_g = 0$ V. The rate of deformation is 0.1 Hz, i.e. the duration of one tip approach-retract cycle is 10 s. This allows acquiring sufficiently large number of points to detect the electromechanical oscillations in the noisy signal. The low-speed of deformation ensures the measurements are carried out in quasi-static conditions.

Supplementary Note 2

AFM photodetector (optical lever) calibration. Prior to nanoindentation experiment, we calibrate the sensitivity of the photodetector in order to accurately measure the AFM tip and nanoribbon deflection. In the AFM setup, a photodetector is used to detect the cantilever deflection and thus the measured deflection values are expressed in volts. In order to convert the measurements from volts to nanometers, the photodetector should be calibrated. This is achieved by performing nanoindentation on the SiO_2 substrate. When being deformed on top of the hard substrate, the deformation of the substrate is negligible compared to the deflection of the cantilever, resulting in a one-to-one correspondence between the deflection of the cantilever and the voltage measured by the photodetector. The sensitivity of photodetector S is then defined as[1]:

$$S\left[nm/V\right] = \frac{Z_{\text{piezo}}[nm]}{D_{\text{volts}}[V]} \tag{1}$$

Where Z_{piezo} is the piezo scanner extension and D_{volts} is the change in the voltage signal on the AFM photodetector due to the deflection of cantilever. For a typical load-deflection calibration curve the sensitivity of the photodetector is calculated from the inverse of the slope of the curve. Supplementary Figure 3 shows the calibration curve for all the devices presented in this work. The slope of load-deflection curve is extracted by fitting a line to it and the corresponding photodetector sensitivity is calculated for each device. Using the calculated sensitivity values makes it possible to determine the cantilever deflection in nanometers.

AFM probe characteristics. We used Mikromasch HQ probes (Model NSC36/AL BS). As reported by the manufacturer, the probe bulk resistivity is in the range of 0.01 Ω·cm to 0.025 Ω·cm. Scanning electron microscopy (SEM) was used to estimate the tip radius (Figure S4). According to SEM image results, the radius of imaged tips vary in the range of 15 nm to 35 nm. The tip height is in the range of 12−18 μm and it has the shape of a three-sided pyramid with the cone angle of 40°, Supplementary Figure 4.

Prior to performing the nanoindentation experiment on each suspended device, the spring constant of the AFM probe is calculated using the Hutter-Bechhoefer method[2] based on acquiring the spectra of thermally induced cantilever motion. The extracted values of

stiffness are in the range of 2 N/m to 7 N/m. Briefly, the energy of the thermally induced vibrations is expressed as:

$$E_{\text{thermal}} = \frac{1}{2} k_{\text{cantilever}} \left\langle D_{\text{thermal}}^2 \right\rangle \quad (2)$$

where $k_{\text{cantilever}}$ is the cantilever spring constant and $\left\langle D_{\text{thermal}}^2 \right\rangle$ is the average of the square of the thermal vibration amplitude of the cantilever. Using the equipartition theorem:

$$\frac{1}{2} k_{\text{cantilever}} \left\langle D_{\text{thermal}}^2 \right\rangle = \frac{1}{2} k_{\text{B}} T^2 \quad (3)$$

By recording the power spectral density of cantilever vibrations (Supplementary Figure 5a), we can obtain $\left\langle D_{\text{thermal}}^2 \right\rangle$ by integrating the area under the resonance peak (Supplementary Figure 5b) and calculate the cantilever spring constant $k_{\text{Cantilever}}$.

Supplementary Note 3

Influence of different boundary conditions. The simplest way of modeling the effect of metallic clamps is to consider them as ideal clamps i.e. the GNRs are fixed and the displacement is zero at the edges. The role of the ideal clamps is simulated by imposing fixed boundary conditions on the two edges of the GNR. The corresponding FEM geometry is illustrated in Supplementary Figure 6a and labeled as “geometry I”.

In another geometry which is one step closer to the realistic devices, one should consider the isotropic nature of HF wet etching meaning that the SiO_2 would be etched in the horizontal direction as much as it would be etched in the vertical direction. This results an undercut in the contact area (visible in the SEM image of Figure 1a. and schematics illustration in Figure 1b.). For instance if the GNR is suspended 180 nm above the substrate, 180 nm of the SiO_2 underneath the contacts is also etched therefore in addition to the GNR, 180 nm of the contacts is also suspended. To model this effect, we use the geometry of Supplementary Figure 6b where the suspended parts of electrodes are left free to deform. This geometry is referred to as “geometry II”.

Finally, the most realistic model takes into account that graphene- SiO_2 interface absorbs HF very fast thus etch away all the SiO_2 below the suspended graphene[3,4]. In the clamping regions, both metal electrode and graphene are suspended and free to deform (illustrated in Figure 1b in the manuscript). The FEM geometry for this model is shown in Supplementary Figure 6c and is labeled as “geometry III”.

In order to find out how different would be to calculate the GNR strain using the three different boundary conditions; we run the simulations for all the GNR devices using the three above mentioned geometries. The GNRs are deformed up to the experimental values corresponding to Figure 2b and Figure 3b of the manuscript. The deformation profile in the three cases are compared and no difference between them is observed. Supplementary Figure 7 shows the simulation results for device #1 using the three different geometries. The results show that the deformation of the metallic clamps remains less than 0.1 nm while the GNR channel is deformed up to 50 nm.

FEM simulations indicate that in our devices, the deformation of the contacts is negligible leading to the same value of strain and deformation as the ideal, rigid clamps. The difference with the results presented in Figure 2 in Huang et al.[5] can be explained considering that they

have used wedge shaped indenters with width bigger or equal to the graphene membrane while we have used sharp AFM tips. The use of wedge shaped wide indenters leads to a uniform strain in the graphene membrane meaning that the region of membrane close to the metal contacts is experiencing as high strain as the part under the indenter. In our experiment, as we have discussed in Supplementary Section 6, the strain distribution is not uniform and most of the strain is concentrated in the vicinity of the AFM tip. Therefore, the areas of GNR close to the metal clamps undergo a much lower strain which is not enough to induce a considerable deformation in the suspended part of contacts.

Supplementary Note 4

Finite element modelling: strain distribution and the gauge factor. For GNRs that are much wider than the AFM tip radius, the sharp AFM tip introduces local strain at the center of the ribbon and the resulting strain distribution is not uniform. In this case, the strain is due to two main contributions. First is dominant in the areas close to the AFM tip due to a local deformation of the ribbon around the sharp, indenting probe. In addition to the non-uniform strain field under the tip, the GNR undergoes a vertical deflection which is the second contribution and is dominant in GNR regions farther away from the AFM tip.

Using finite element modelling (FEM), we simulated the strain distribution on the GNRs of devices #1-#7 in order to determine the maximal strain. Each device is modelled as a suspended clamped GNR. For each device, the geometry is extracted from AFM images and each device is deformed to the same value of deformation corresponding to the data shown in Figures 2b and 3b in the main manuscript. The nominal thickness of 0.335 nm and 0.69 nm have been used for single layer and double layer devices, respectively. Young’s modulus of 1 TPa, Poisson’s ratio of 0.165 and linear elastic material was assumed as the parameters. A linear elastic behavior is assumed since in the data presented in manuscript, the maximum applied strain is 4% and it has been shown by Lee. et al.[6] that only for strain higher than threshold of 5% the non-linear behavior starts to become considerable. Initially the clamped edges of the ribbon were modelled by imposing fixed constraint boundary condition. Other possibilities for boundary conditions are also considered which is discussed in details in Supplementary Section 7. The AFM tip was modelled as a rigid sphere and is indenting the GNR in the center up to a predefined displacement value which is set as an input parameter to the model. Contact between the membrane and the indenter is modelled as a frictionless contact with the contact pressure described using Augmented Lagrangian method. Using the stationary study, we determined the strain distribution for each suspended device when it is deformed to the values corresponding to the experimental measurements.

Figure S6 shows the 2D map of the strain distribution on two of the narrowest ribbons (Devices #1 and #3) and two of the widest ribbons (Devices #6 and #7). As illustrated in Supplementary Figure 8, the strain distribution map on GNRs can be described by a combination of two different effects. The first effect is the local deformation of the GNR in the vicinity of the AFM tip. The sharp tip of AFM introduces a local strain which can reach strain values as high as 4% for the deformation values corresponding the data shown in Figures 2b and 3b in the manuscript. The second effect is the vertical deflection of the GNR which forms two segments of straight lines. This effect is more prominent for the regions farther away from the center, where the strain is uniformly distributed on the GNR and the strain level determined by FEM simulations are equal with the strain levels calculated by the expression for uniform
strain ε:

$$\varepsilon = \frac{L - L_0}{L_0} = \frac{2\sqrt{\left(\frac{L_0}{2}\right)^2 + D_{\mathrm{GNR}}^2} - L_0}{L_0} \tag{4}$$

where L is the length of the strained GNR, L_0 its relaxed length and D_{GNR} the GNR deflection at mid-point. While both effects are present in all of the GNRs, the FEM simulations indicate that for GNRs with widths closer to the tip radius, the global vertical deformation dominates in areas away from the tip. This observation is expected since the ratio between the GNR width and the tip diameter determines how uniformly the tip could deflect the GNR.

The strain profile along the dashed lines in Supplementary Figure 8a - d is shown in Supplementary Figure 9a - d. Device #3 with the width of 60 nm is the narrowest GNR measured. The strain distribution for this device (Supplementary Figure 9b) clearly shows a plateau of constant strain of 1.7% in regions far from the AFM tip. This value is equal to the strain calculated using Supplementary Equation 4 for a GNR of initial length L_0 = 650 nm and D_{GNR} = 60 nm (deflection of Device #3 data in Supplementary Figure 8b). For the same device, Supplementary Figure 9b shows that in the region of GNR which is closer to the AFM tip the strain is not constant and is increasing up to 4% which is due to the local deformation effect of the AFM tip. Comparing the four GNRs presented in Supplementary Figure 9, one conclusion is that for wider GNRs, the plateau of constant strain is smaller and the non-uniformity of the strain distribution is more pronounced.

Using finite element modeling, we can estimate the upper limit on the gauge factor GF defined as:

$$GF = \frac{\Delta R / R_0}{\varepsilon} \tag{5}$$

where R_0 is the electrical resistance of the undeformed material and ΔR the change in resistance under the application of strain ε. The upper limit of gauge factor could be calculated by taking into account only the second contribution to strain, related to the vertical deflection of the GNR and corresponding to the plateau of constant strain (Supplementary Figure 9). The electromechanical behavior of device #1 to device #4 shows that the gauge factor of our single layer GNRs is positive and smaller than 8.8 and comparable to previously reported values[7,8] In comparison, the piezoresistive gauge factor of atomically thin MoS_2, an emerging semiconducting two dimensional material, is shown to be more than 200[9], the gauge factor of silicon reaches ~200[10] and for single-wall carbon nanotubes it can be as high as ~2900[11,12].

Supplementary Note 5

Mechanical failure and highest achieved strain. Each suspended GNR was deformed in several extension-retraction cycles with the extension depth increasing successively until the mechanical failure of the device is observed. Supplementary Figure 10 shows the electromechanical response of devices #2-5 at the mechanical failure. All devices show

similar electromechanical behavior. Before the mechanical failure occurs, the current I_d decreases with the increasing deformation. Once the suspended structure fails (the point marked by black arrow), the cantilever deflection drops sharply and the current I_d drops to zero. By further extension of the piezo scanner Z_{piezo} the cantilever starts deforming on top of the hard substrate, thus the linear shape of the $D_{cantilever}$ vs. Z_{piezo} curve. During the retraction cycle, the tip starts retracting from the hard substrate with the same linear shape for the $D_{cantilever}$ vs. Z_{piezo} curve. The current I_d remains zero as the GNR channel has failed. Using FEM the maximum achieved strain is calculated to be 5%.

Supplementary Note 6

Reproducibility of electromechanical oscillations. Repeated experiments have been conducted in order to ascertain the reproducibility of the observed electromechanical oscillations for bilayer GNRs. Two samples from distinct fabrication batches have been probed. For each device, several consecutive nanoindentation cycles are performed. Results from an additional bilayer graphene device show reproducible electromechanical oscillations through successive cycles of loading-unloading with increasing strain. For these successive deformations, current oscillations overlap almost perfectly with slight shifts appearing at high strains. This confirms the reproducibility of the measurements.

Supplementary Note 7

Estimation of the domain boundary width. Below, we estimate the width of the transition region W for the case of large ΔW characterized by the formation of a ripple in graphene layer with area excess. We describe the ripple by a sinusoidal out-of-plane deformation of form

$$\xi(x) = \frac{A}{2}\sin\frac{2\pi x}{W} \tag{6}$$

Hence, the amplitude of the out-of-plane deformation

$$A = \frac{2W}{\pi}\sqrt{\frac{\Delta W}{W}} \tag{7}$$

The energy of the domain boundary E per unit length results from two contributions, $E = E_1 + E_2$. The first contribution represents the binding energy of the two layers lost upon delamination due to the ripple formation

$$E_1 = \varepsilon_b W \tag{8}$$

where is $\varepsilon_b = 1.83$ eV/nm^2 is the binding of graphene layers energy per unit area.[13] The second contribution originates from the bending of the rippled layer. The bending energy is given by[14]

$$E_2 = \frac{1}{2}\kappa\left\langle(\xi''_{xx})^2\right\rangle W = 4\kappa\pi^2\frac{\Delta W}{W^2} \tag{9}$$

where κ = 1.42 eV is the bending rigidity of graphene.

The actual value transition region width W minimizes the energy of domain boundary E, hence

$$W = 2\sqrt[3]{\frac{\kappa\pi^2 \Delta W}{\varepsilon_\mathrm{b}}} \tag{10}$$

For a typical path difference ΔW = 1 nm considered by our simulations the domain boundary width $W \approx$ 4 nm. This justifies the assumption of ballistic transmission across the investigated domain boundaries in bilayer graphene.

Supplementary Note 8

Simulations of electronic transport across domain boundaries in bilayer graphene. We use a tight-binding model of bilayer graphene with the nearest-neighbor hopping term within each graphene layer, and the interlayer hopping being restricted only to the sites aligned along the direction normal to the plane. The Hamiltonian can be written as[15]

$$H = -t \sum_{m,\langle i,j\rangle} \left(a_{mi}^\dagger b_{mj} + \text{h. c.}\right) - t_\perp \sum_i \left(a_{1i}^\dagger a_{2i} + \text{h. c.}\right) \tag{11}$$

where $a_{mi}\left(b_{mi}\right)$ annihilates an electron in graphene layer m = 1,2 at site $\mathbf{R}_i$ belonging to sublattice A(B) , t = 2.66 eV is the intralayer nearest-neighbor hopping energy and $t_\wedge$ = 0.4 eV is the interlayer hopping energy.

Based on the Landauer-Büttiker formalism[16,17], we first compute the ballistic transmission probability T as a function of momentum $k_{||}$ and energy E

$$T(k_{||},E) = \mathrm{Tr}\left[\Gamma_\mathrm{L}(k_{||},E)G_\mathrm{S}^\dagger(k_{||},E)\Gamma_\mathrm{R}(k_{||},E)G_\mathrm{S}(k_{||},E)\right] \tag{12}$$

using the Green's function

$$G_\mathrm{S}(k_{||},E) = \left[E^+ I - H_\mathrm{S} - \Sigma_\mathrm{L}(k_{||},E) - \Sigma_R(k_{||},E)\right]^{-1} \tag{13}$$

where I is a unitary matrix, $E^+ = E + i\eta \ (\eta \to 0)$, and H_S is the Hamiltonian for the scattering region. The coupling matrices are given by

$$\Gamma_\mathrm{L(R)}(k_{||},E) = i\left[\Sigma_\mathrm{L(R)}(k_{||},E) - \Sigma_\mathrm{L(R)}^\dagger(k_{||},E)\right] \tag{14}$$

where $\Sigma_\mathrm{L(R)}$ are the self-energy matrices of bilayer graphene leads.

Finally, the conductivity G is given by

$$G = G_0 \sum_{k_{||},\,E} T(k_{||},\, E) \tag{15}$$

where $G_0 = \dfrac{e^2}{h}$ is the quantum of conductance.

Supplementary Figure 11 shows a schematic drawing of a typical model employed for studying the discussed transport phenomena. Semi-infinite AB-stacking bilayer graphene leads are separated by the scattering region in which the two graphene layers are locally decoupled and which incorporates an effective width difference ΔW. The structure is periodic along the armchair direction while transport is simulated along the zigzag direction. It was shown that transport properties of such model systems are insensitive to the overall width of the scattering region and the only relevant parameter is the effective width difference ΔW.

Supplementary References

1. Hu, Z., Seeley, T., Kossek, S. & Thundat, T. Calibration of optical cantilever deflection readers. *Rev. Sci. Instrum.* **75,** 400–404 (2004).
2. Hutter, J. L. & Bechhoefer, J. Calibration of atomic‐force microscope tips. *Rev. Sci. Instrum.* **64,** 1868–1873 (1993).
3. Bolotin, K. I. *et al.* Ultrahigh electron mobility in suspended graphene. *Solid State Commun.* **146,** 351–355 (2008).
4. Chen, C. *et al.* Performance of monolayer graphene nanomechanical resonators with electrical readout. *Nat. Nanotechnol.* **4,** 861–867 (2009).
5. Huang, M., Pascal, T. A., Kim, H., Goddard, W. A. & Greer, J. R. Electronic−Mechanical Coupling in Graphene from in situ Nanoindentation Experiments and Multiscale Atomistic Simulations. *Nano Lett.* **11,** 1241–1246 (2011).
6. Lee, C., Wei, X., Kysar, J. W. & Hone, J. Measurement of the elastic properties and intrinsic strength of monolayer graphene. *Science* **321,** 385–8 (2008).
7. Huang, M., Pascal, T. A., Kim, H., Goddard, W. A. & Greer, J. R. Electronic−Mechanical Coupling in Graphene from in situ Nanoindentation Experiments and Multiscale Atomistic Simulations. *Nano Lett.* **11,** 1241–1246 (2011).
8. Smith, A. D. *et al.* Electromechanical Piezoresistive Sensing in Suspended Graphene Membranes. *Nano Lett.* **13,** 3237–3242 (2013).
9. Manzeli, S., Allain, A., Ghadimi, A. & Kis, A. Piezoresistivity and Strain-induced Band Gap Tuning in Atomically Thin MoS_2. *Nano Lett.* (2015).
10. Kanda, Y. Piezoresistance effect of silicon. *Sens. Actuators Phys.* **28,** 83–91 (1991).
11. Sickert, D. *et al.* Strain sensing with carbon nanotube devices. *Phys. Status Solidi B* **243,** 3542–3545 (2006).
12. Stampfer, C. *et al.* Nano-Electromechanical Displacement Sensing Based on Single-Walled Carbon Nanotubes. *Nano Lett.* **6,** 1449 – 1453 (2006).
13. Lebègue, S. *et al.* Cohesive Properties and Asymptotics of the Dispersion Interaction in Graphite by the Random Phase Approximation. *Phys. Rev. Lett.* **105,** 196401 (2010).
14. Yazyev, O. V. & Louie, S. G. Electronic transport in polycrystalline graphene. *Nat. Mater.* **9,** 806–809 (2010).
15. Castro Neto, A. H., Guinea, F., Peres, N. M. R., Novoselov, K. S. & Geim, A. K. The electronic properties of graphene. *Rev. Mod. Phys.* **81,** 109–162 (2009).
16. Büttiker, M., Imry, Y., Landauer, R. & Pinhas, S. Generalized many-channel conductance formula with application to small rings. *Phys. Rev. B* **31,** 6207–6215 (1985).
17. S. Datta. *Electronic transport in mesoscopic systems*. (Cambridge University Press, 2007).
